# Spatial and temporal variation of routine parameters: pitfalls in the cerebrospinal fluid analysis in central nervous system infections

**DOI:** 10.1186/s12974-022-02538-3

**Published:** 2022-07-06

**Authors:** Marija Djukic, Peter Lange, Frank Erbguth, Roland Nau

**Affiliations:** 1 Institute of Neuropathology, University Medical Center, Göttingen, Germany; 2 Department of Geriatrics, Protestant Hospital Göttingen-Weende, An der Lutter 24, 37075 Göttingen, Germany; 3 Department of Neurology, University Medical Center, Göttingen, Germany; 4 Paracelsus Medical Private University, Nuremberg, Germany

**Keywords:** Cerebrospinal fluid, Blood–CSF barrier, Blood–brain barrier, Lactate, Intrathecal immunoglobulin synthesis, CSF flow

## Abstract

The cerebrospinal fluid (CSF) space is convoluted. CSF flow oscillates with a net flow from the ventricles towards the cerebral and spinal subarachnoid space. This flow is influenced by heartbeats, breath, head or body movements as well as the activity of the ciliated epithelium of the plexus and ventricular ependyma. The shape of the CSF space and the CSF flow preclude rapid equilibration of cells, proteins and smaller compounds between the different parts of the compartment. In this review including reinterpretation of previously published data we illustrate, how anatomical and (patho)physiological conditions can influence routine CSF analysis. Equilibration of the components of the CSF depends on the size of the molecule or particle, e.g., lactate is distributed in the CSF more homogeneously than proteins or cells. The concentrations of blood-derived compounds usually increase from the ventricles to the lumbar CSF space, whereas the concentrations of brain-derived compounds usually decrease. Under special conditions, in particular when distribution is impaired, the rostro-caudal gradient of blood-derived compounds can be reversed. In the last century, several researchers attempted to define typical CSF findings for the diagnosis of several inflammatory diseases based on routine parameters. Because of the high spatial and temporal variations, findings considered typical of certain CNS diseases often are absent in parts of or even in the entire CSF compartment. In CNS infections, identification of the pathogen by culture, antigen detection or molecular methods is essential for diagnosis.

## Introduction

In the last decades, many efforts were made to establish etiologic diagnoses in central nervous system (CNS) infections by cerebrospinal fluid (CSF) routine parameters [e.g., 1–6]. However, none were sensitive and specific enough for effective clinical decision-making, either due to rapid temporal variations (e.g., low leukocyte concentrations in the CSF of patients with early bacterial meningitis), rapid resolution of inflammation during adequate antibiotic treatment, or spatial variations (e.g., lumbar versus ventricular CSF) of the parameters studied. In neonatal meningitis caused by *Streptococcus agalactiae* (*n* = 146), CSF routine parameters were even found to be normal in 6% of reported patients [7]. In a meningococcal meningitis cohort (*n* = 258), the CSF leukocyte count was below 1000/µl in 20% of the cases while in 1.7% the CSF leukocyte count was reported as normal [8]. In a group with pneumococcal meningitis (*n* = 153), 17% of the patients had a CSF leukocyte count < 100/µl and 5% < 10/µl [9]. In a case series of *Listeria* meningitis (*n* = 62), CSF findings in 26% of the patients were not typical for bacterial meningitis [10].

Our interest in this topic stems from an actual case at our clinic: one of our patients died of cardiac arrest 2 months after initiation of antibiotic therapy for bacterial meningitis. Three days before her death, lumbar CSF contained 12 leukocytes/μl, a total protein content of 814 mg/l, a CSF lactate of 3.2 mmol/l, a CSF-to-serum albumin ratio of 9.7 × 10^–3^, and an intrathecal IgG, IgA and IgM synthesis. We were convinced that her treatment had been successful, yet at autopsy, a thick layer of pus was found over the convexities and at the bottom of the forebrain [11].

**Fig. 1:**
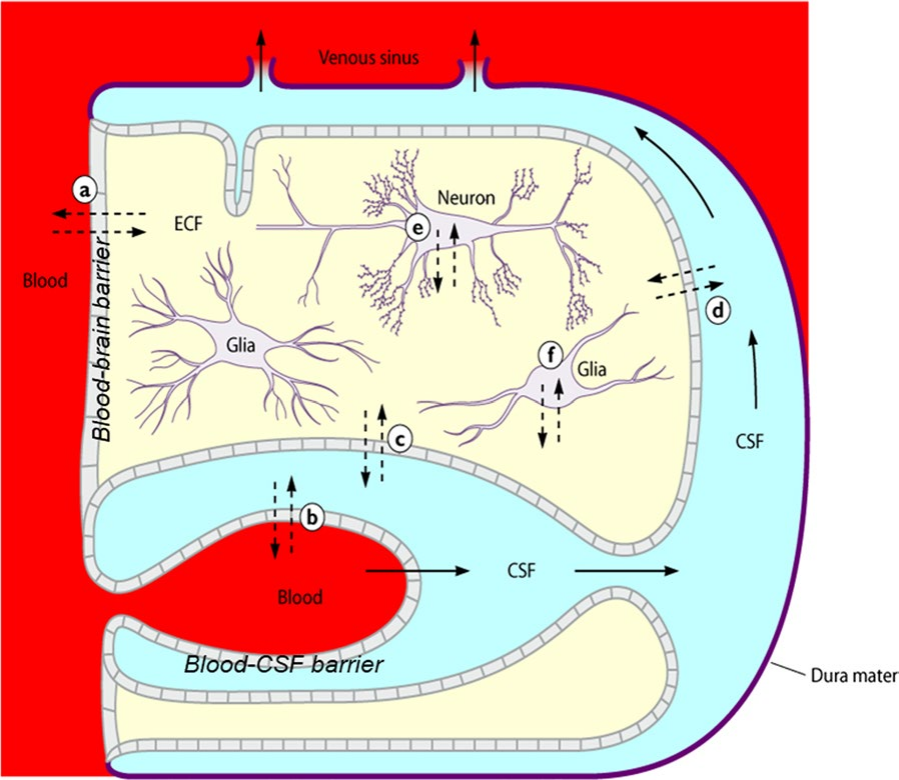
Schematic drawing of the main compartments of the CNS. CSF flow and diffusion of water or solutes in the CNS [12]. Solid line arrows: CSF flow. Dotted arrows: diffusion of water or solutes occurring between brain capillaries, CSF, and nervous tissue **a** across the blood–brain barrier; **b** across the epithelium of the choroid plexus; **c** across the ventricular ependyma; **d** across the pia–glial membranes at the surface of the brain and spinal cord, and **e** and **f** across the cell membranes of neurons and glial cells. (Reproduced from [12, 13] with kind permission of Elsevier HCM - Health Care Management and the American Society for Microbiology.)

**Fig. 2:**
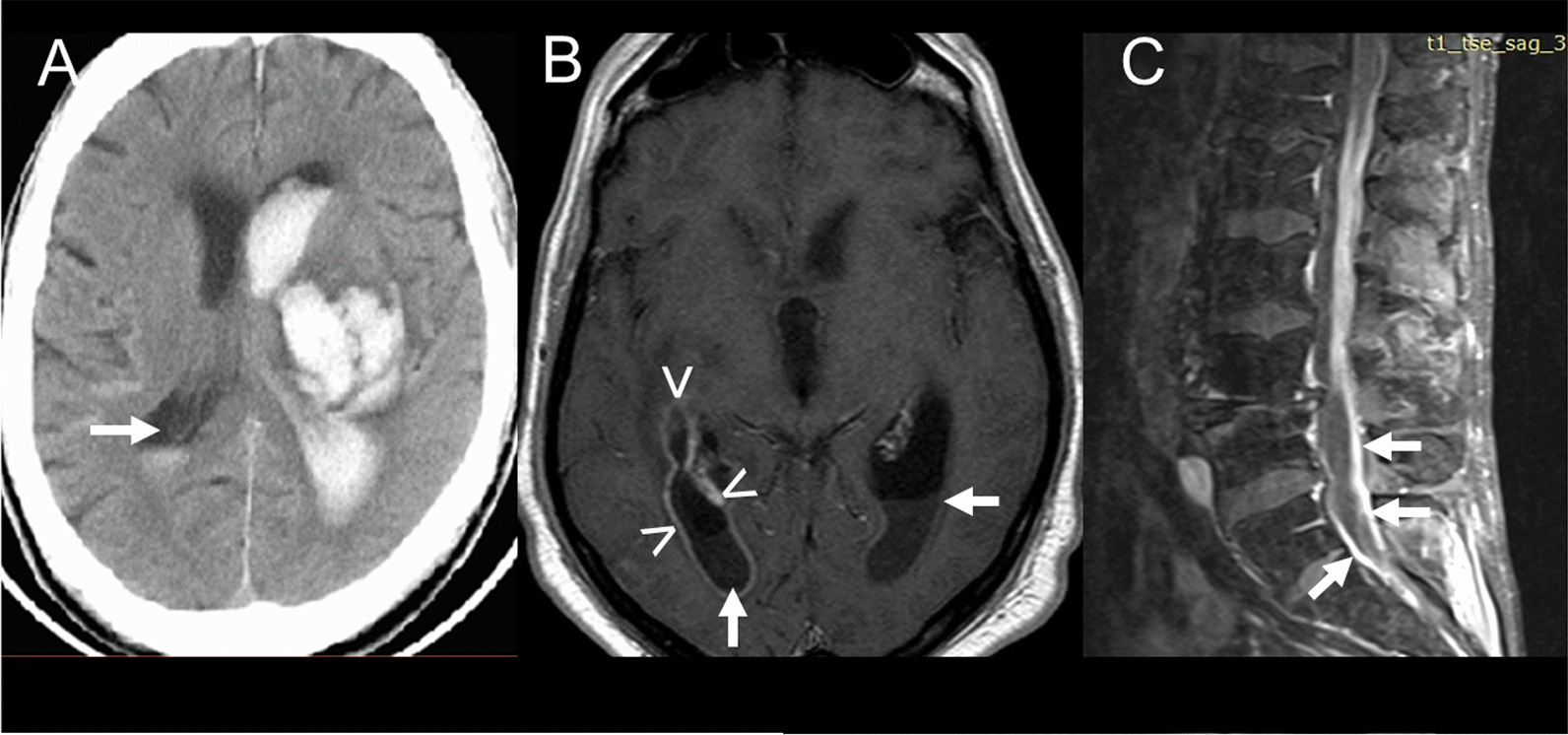
Sedimentation of cells in different parts of the ventricular system visible in clinical routine imaging. **A** Blood sedimentation in the 2nd ventricle (arrow) after intracerebral and intraventricular hemorrhage (cranial computer tomography) (kindly provided by Prof. Dr. Hilmar Prange, Dept. of Neurology, University Medicine Göttingen, Germany). **B** Pus in the dorsal horns of the 1st and 2nd ventricle (arrows), contrast enhancement of the wall of the right lateral ventricle (arrowheads) (Streptococcus intermedius meningitis and ventriculitis; T1-weighted magnetic resonance image plus gadolinium contrast enhancement). **C** Pus in the lumbar spinal canal (arrows) (*Candida albicans* meningitis; T1-weighted magnetic resonance image plus gadolinium contrast enhancement) (kindly provided by Dr. Hans-Heino Rustenbeck, Dept. of Neuroradiology, University Medicine Göttingen, Germany)

The CNS compartments including the CSF space, the extracellular fluid (ECF) of the brain and spinal cord and the intracellular fluids (ICF) of the different intracellular compartments of the various cell types comprising the nervous tissue are separated from the systemic circulation by the blood–brain and blood–CSF barrier (Fig. 1). These barriers protect the central nervous system (CNS) from endogenous and exogenous compounds present in the systemic circulation and are essential to ensure the proper function of the CNS. Concentrations of individual endogenous and exogenous compounds differ between the individual compartments [12, 13]. Moreover, the ECF of the brain and spinal cord is not a homogeneous compartment [14], and the composition of the ICF in the different intracellular compartments of the various cell types depends not only on the cell type, but also on the location of the cell in the CNS [15]. With methods established in clinical medicine [CSF analysis, computer tomographic (CT) or magnetic resonance imaging (MRI), positron emission tomography (PET), in critically ill patients also microdialysis] spatial and temporal variations of the concentrations of endogenous and exogenous compounds are only partly detectable. The inhomogeneity of the CSF space can be visible in CT or MRI images of the brain in subarachnoid hemorrhage or bacterial meningitis cases in which the sedimentation of erythrocytes or leukocytes, respectively, can be observed in either the dorsal parts of the ventricles or in the lumbar CSF space (Fig. 2).

In routine CSF analysis, some parameters (CSF leukocyte count, CSF differential cell count, CSF glucose, CSF/serum glucose ratio) have static cut-off values, i.e., spatial variations are not taken into consideration. For others parameters (CSF protein, CSF/serum albumin ratio, several markers of nervous tissue destruction) attempts have been made to introduce CSF/serum ratios or/and fixed correction factors to account for spatial variation of the concentrations measured in different parts of the CSF space [12, 16–18].

Spatial variations of parameters measured in CSF or microdialysate are difficult to study in humans. Since a lumbar—and even more a cisternal or ventricular—puncture and the implantation of microdialysis fibers are invasive procedures, repeated sampling of CSF or sampling of CSF or microdialysate from different regions of the CSF space is only indicated in exceptional circumstances.

The present review and analysis of previously published data will focus on the CSF compartment as the most easily accessible compartment of the CNS. It aims to assess the utility of generally accepted normal values in CSF analysis as well as providing an aid for interpreting CSF findings in various diseases.

## Anatomy and pathophysiology

The blood–brain and blood–CSF barrier can be viewed simply as a single (the tight junctions between the cells) or double lipid layer (the cells consisting of a basal and apical cell membrane and the cytoplasm in between) surrounding the CNS with leaky regions (gap junctions instead of tight junctions) comprising approximately 1:5000 of the entire capillary surface area of the CNS [19].

The CSF space is convoluted. This convolution and the CSF flow preclude rapid equilibration between the different parts of the compartment. Briefly, the CSF space comprises the four ventricles, the aqueduct, the basal cisterns, and the subarachnoid space over the convexities and in the spinal canal. There is high interindividual variation in the size of the CSF space. The volume of the CSF varies widely depending on age, genetic and environmental factors, underlying diseases, ventricular volume, volume of the cerebral subarachnoid space, individual height, sex, as well as the width of the spinal canal. MRI studies estimating the CSF volume in healthy volunteers showed a cranial CSF volume of approximately 96 ml in young children (19–33 months), approximately 250 ml in middle-aged adults (40–55 years), and approximately 300 ml in the elderly (71–80 years) [20, 21]. In communicating hydrocephalus and non-communicating hydrocephalus the intracranial CSF volume is approximately 500 ml [22]. Blood clots in the ventricle(s) or basal cisterns or a mass lesion or brain edema can substantially diminish the volume of intracranial CSF. The volume of CSF in the spinal canal is also highly variable and is primarily dependent on the individual`s height and the width of the spinal canal. It ranges from 30–80 ml [12, 23]. The relations between the CSF flow rate and the CSF volume, dependent on all the determinants listed, influence the CSF protein content and the CSF-to-serum albumin concentration ratio (*Q*_Alb_) in the different parts of the CSF space [24–26].

As outlined in the Introduction, even under physiological conditions, the distribution of compounds in the CSF is not homogeneous [11, 27–29]. Under pathological conditions, obstacles further impeding the distribution of molecules and cells in the CSF are frequent: intraventricular hemorrhage or tumor, obstruction of the foramina of Luschka and Magendie or of the basal cisterns impeding the communication of CSF between the 4th ventricle and the cerebral convexities and the spinal canal, stenoses of the cervical, thoracal or lumbar spinal canal as well as accumulation of pus in the ventricles, over the convexities or in the spinal canal (Fig. 2).

Approximately two-thirds of the CSF is produced by the choroid plexuses of the ventricles, and about one-third stems from the extracellular space of brain and spinal cord. The CSF flow oscillates, depending on heartbeats, head movements and respiration. These oscillations promote the equilibration of molecules and cells in the CSF space. There is a net flow from the ventricles to the Cisterna magna. From there, the CSF flow divides into the cerebral convexities and the spinal canal [12, 30–33]. The coordinated action of the cilia of the plexus ependyma maintains a network of fluid flows in the 3rd ventricle of the mouse, which allows precise CSF transport. A cilia-based switch can periodically alter the CSF flow pattern which may control distribution of endogenous and exogenous compounds in the 3rd ventricle [33, 34]. Complex flow patterns were also present in the third ventricles of rats and pigs [33], suggesting that ciliated epithelia can generate and maintain complex, spatiotemporally regulated flow networks.

The CSF production rate (equivalent to the rate of CSF bulk flow) is not constant. In a study of six normal volunteers, a circadian rhythm was noted with a minimum production of 30% of the maximum values (12 ± 7 ml/h) at about 6:00 p.m. with a peak production of 42 ± 2 ml/h at night, at about 2:00 a.m. [35]. In Alzheimer’s disease, CSF production is reduced to about 50% of the normal values [36]. Inter- and intra-individual variations similar to healthy persons were found in a study of patients with external ventriculostomies: in 12 of these patients (age 30–69 years), the mean CSF flow rate out of the drain (± SD) was 7.5 ± 3.4 ml/h (range 1.6–12.1 ml/h) [37]. Several drugs commonly used in clinical medicine can inhibit the CSF production: furosemide, acetazolamide, spironolactone, amiloride, digitalis glycosides, vasopressin, corticosteroids, osmotic agents and non-steroidal anti-inflammatory drugs [12, 38]. The modulation of CSF production by blood and intracranial pressure is considered to be low to moderate [12, 39].

In contrast to the blood–CSF and blood–brain barrier, the CSF and the extracellular fluid of the brain and spinal cord are not tightly separated. Even large molecules up to the size of horseradish peroxidase can enter the cerebral extracellular space from the CSF [40]. According to different estimates, under physiological conditions the extracellular space of the brain comprises 15–20% of total brain volume [12, 30]. It has been hypothesized that during occlusive hydrocephalus as well as in the presence of intracranial mass lesions, the brain behaves like a sponge, adjusting to compression by displacing fluid from its veins and the ECF [41, 42]. Molecules from CSF enter the ECF of the brain and spinal cord by diffusion, against the direction of CSF bulk flow, from the nervous tissue into the CSF [12].

## Distribution of compounds in the CSF space—what we have learned from drugs and radiolabelled natural compounds

The following summarizes key publications on the distribution of hydrophilic drugs in the CSF after different modes of injection directly into the lumbar CSF or the ventricles. Since small lipophilic drugs readily cross the blood–brain and blood–CSF barrier from CSF to blood, they are unsuitable to study distribution in the different parts of the CSF compartment. Therefore, data on the pharmacokinetics of small lipophilic drugs [e.g., the anticancer agent triethylenephosphoramide (thiotepa)] after intrathecal administration are not included here [43, 44].

### Intravenous infusion/injection

After an intravenous injection, the time it takes to reach equilibrium between blood and CSF depends on the molecular mass and lipophilicity of the compound studied and on the size of the host organism: ions and small moderately lipophilic drugs rapidly equilibrate, whereas large hydrophilic compounds need up to several days to fully equilibrate [45–47]. Equilibrium is reached more rapidly in the ventricles and the cisterna magna than in the lumbar sac [47], and small animals have a quicker CSF turnover than humans [12]. For drugs, the ratio of the CSF and serum concentration at steady state (*C*_CSF_/*C*_S_) and the ratio of the areas under the concentration–time curves in CSF and serum (AUC_CSF_/AUC_S_) after a short-duration infusion depend on the molecular mass, lipophilicity and binding to plasma proteins of the compound studied as well as on the state of the blood–CSF barrier and the CSF flow [12, 13, 48]. Again, *C*_CSF_/*C*_S_ and AUC_CSF_/AUC_S_ are higher in the lumbar than in the ventricular CSF leading to higher lumbar than ventricular drug concentrations. With the exception of small, moderately lipophilic drugs, elimination from the blood is faster than elimination from the CSF, resulting in a lag in the concentration–time curve in CSF in comparison to the respective curve in serum [13, 48].

In humans, after an intravenous infusion of radiolabeled albumin, it takes approximately 3 days to establish an equilibrium between blood and CSF, due to the dynamic exchange of albumin between these two compartments [45]. The entry of radiolabeled immunoglobulins from the blood into the CSF was even slower. Here, equilibrium was reached after approximately 4 days [46]. When radiolabeled serum albumin was administered intravenously to infants with communicating hydrocephalus, 4–9 h later radioactivity was highest in lumbar fluid, intermediate in cisternal and lowest in ventricular fluid [49].

### Intralumbar injection does not ensure distribution into the ventricles

Injection into the lumbar CSF does not ensure a homogeneous drug distribution in the whole CSF space: after intralumbar injection, the drug concentrations in ventricular CSF are highly variable. Often, therapeutic concentrations are not achieved [50–52]. After lumbar injection of 5–10 mg gentamicin or tobramycin in infants, the concentrations of both aminoglycosides in the cisternal CSF were always lower than those in lumbar CSF and peaked at 14 h. Gentamicin and tobramycin levels in the ventricular CSF ranged from 0 to 2.1 mg/l and often were not therapeutic [50]. Compared to the supine, the prone position for 60 min promoted methotrexate entry into the ventricular CSF after lumbar administration [53].

### Intraventricular injection leads to rapid distribution in the whole CSF space

Unless there is a complete block of the CSF circulation, e.g., in cerebral aqueduct stenosis or after an obstruction of the cervical, thoracic or lumbar spinal canal, intraventricular drug injection ensures its distribution throughout the entire CSF space. Peak aminoglycoside concentrations in lumbar CSF were 2–10 times lower than the simultaneous intraventricular levels, but still in the therapeutic range [51]. In infants, drug concentrations in the ventricular and lumbar CSF were almost equal 2–48 h after injection of 5 mg gentamicin or tobramycin into one lateral ventricle [50].

### Injection into one lateral ventricle does not always lead to equal concentrations in the other lateral ventricle

In neonates with bacterial meningitis, injection of amikacin into one ventricle resulted in approximately equal drug levels in both ventricles 10 h after dosing [51]. Conversely, in a hydrocephalic 64-year-old male patient suffering from *Scedosporium apiospermum* meningitis, daptomycin concentrations measured in the CSF from the right and left ventricle were discordant by a factor of about 3 after injection of daptomycin into the right ventricle [54].

### Elimination

Elimination from the CSF compartment occurs through: (a) CSF bulk flow with peripheral outflow of CSF along all nerves and (possibly only during conditions of increased intracranial pressure) by the arachnoid granulations; (b) diffusion across the blood–brain and blood–CSF barrier [12, 13, 48, 55], and (c) bidirectional fluid exchange between the Virchow–Robin spaces surrounding vessels penetrating into the brain parenchyma and the brain extracellular fluid, the subarachnoid CSF space and the venous perivascular space [56]. The extracranial portions of cranial nerves and the regions of the spinal nerves close to the dorsal root ganglions are the only routes where in morphological studies barriers at the arachnoid or endothelial cell layers were lacking, allowing bulk outflow shown in physiological studies [55]. In ex vivo studies, the arachnoid granulations allow the passage of large molecules, bacteria and even particles up to a size of 7.5 μm [57]. Anatomical studies, however, have failed to demonstrate structures extending from the CSF space to the dural sinuses without intact leptomeningeal layers lying in between [58]. At present, there is little support for an outflow of CSF via the arachnoid granulations under normal conditions, however, this route may become recruited during conditions of increased intracranial pressure [55]. Erythrocytes are unable to pass the blood–CSF and blood–brain barrier, and the passage of leukocytes through these anatomical structures is an active migratory process [59]. The contribution of dural lymphatic vessels originally described in 1787 and rediscovered in 2015 [60–62] to the drainage of CSF is a matter of debate. The arachnoid barrier between the subarachnoid space and interstitial tissue of the dura mater (layers of closely apposed, epithelial-like cells connected by complexes of tight junctions) makes it very unlikely that these vessels contribute to CSF bulk flow [55, 63]. Taken together, large molecules and small particles are mainly eliminated from the CSF by bulk flow. After intraventricular injection, elimination of the hydrophilic antibiotic amikacin was slower in hydrocephalic patients than in patients with a normal ventricle size [51]. With small and/or lipophilic molecules and/or molecules with a high affinity to efflux pumps bulk flow is less important, since retrograde diffusion across the blood–brain and blood–CSF barrier and/or active transport predominates [64]. Of the parameters used in routine CSF analysis, only lactate is small enough to diffuse across the barriers in a retrograde manner at clinically relevant degrees, and glucose crosses the blood–brain barrier by facilitated diffusion [65].

In humans, an early estimate of the CSF turnover rate was approximately 22%/h [12] corresponding to an elimination half-life t_1/2β CSF_ of 3.15 h. However, in view of recent data on the CSF production rate and the volume of the CSF space, this estimate appears to be too high. Assuming an average production of 20 ml of CSF/h and an average CSF volume of 200 ml would lead to an estimated CSF turnover rate (i.e., the elimination rate constant k_el_) of 0.1/h (10%/h) and of t_1/2β CSF_ of 6.9 h. The average time a “γ-globulin molecule” spent in human CSF was estimated to be 17.3 h which, when assuming first order kinetics, corresponds to a t_1/2β_ of 12.0 h [66]. After intraventricular injection of antibiotics in humans, the measured t_1/2β CSF_ of large hydrophilic antibiotics correspond well to this estimate: t_1/2β CSF_ of vancomycin (molecular mass 1486 g/mol) ranges from approximately 2 to 20.5 h [52, 67–72]. T_1/2β CSF_ of colistin (molecular mass 1155 g/mol) after intraventricular administration in humans [73] was similar to that of vancomycin (7.8 ± 3.2 h). Gentamicin and tobramycin, also hydrophilic, but with slightly smaller molecular masses (478 and 468 g/mol), showed a CSF elimination half-life of 6.2–6.4 h [50]. The high variation of t_1/2β CSF_ estimated in clinical studies in critically ill patients originates from several variables: (1) inter- and intra-individual differences of the CSF volume, (2) inter- and intra-individual variations of the CSF production and absorption, and (3) the rate of CSF drainage by an external or internal ventricular catheter [72, 73]. Generally, the elimination half-life (t_1/2 β_) of hydrophilic molecules in the CSF after intrathecal administration shows a great interindividual variation and in most cases lies between 6 and 12 h.

## How to estimate blood contamination of CSF samples

**Fig. 3:**
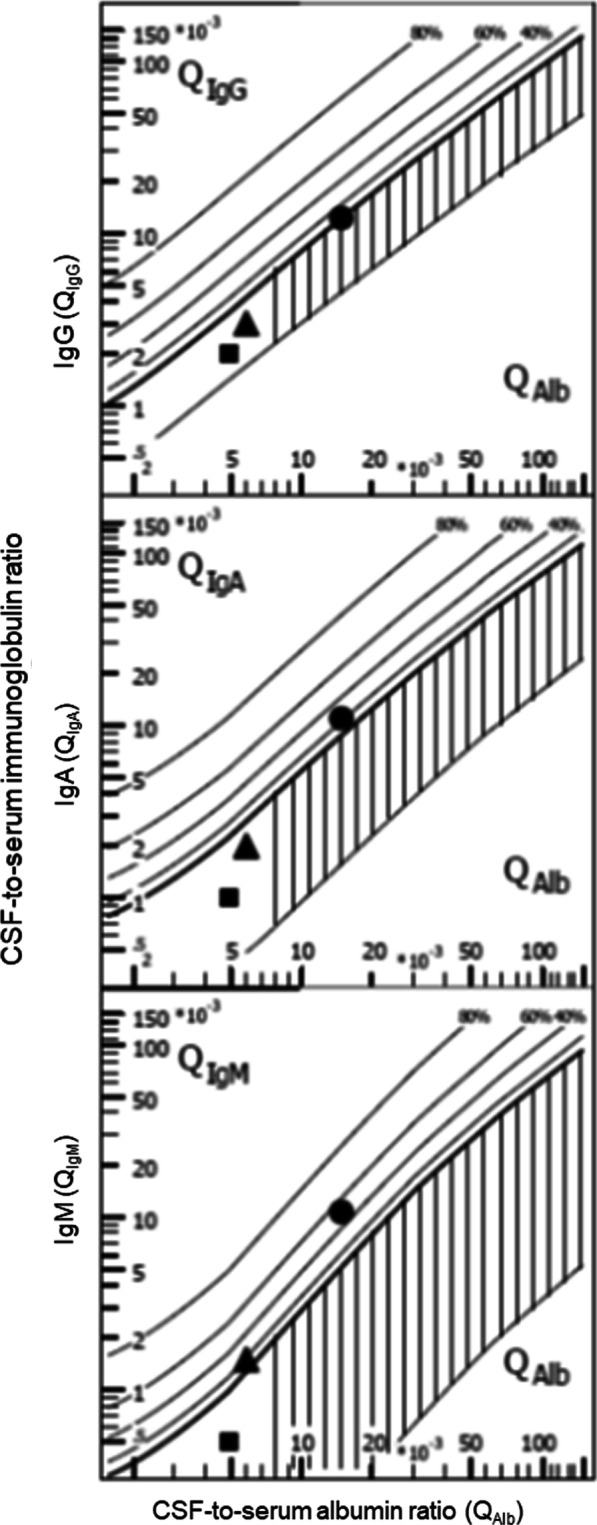
Influence of blood contamination of the CSF on Reiber–Felgenhauer nomograms—hypothetical cases. Contamination of CSF by blood can falsify a finding of intrathecal immunoglobulin synthesis, as estimated by Reiber–Felgenhauer nomograms. To illustrate this fact, we performed the following model calculation. In samples without blood contamination (filled squares) we calculated with the following concentrations: albumin CSF 200 mg/l, serum 40,000 mg/l; IgG CSF 20 mg/l, serum 10,000 mg/l; IgA CSF 2 mg/l, serum 2000 mg/l; IgM CSF 1 mg/l, serum 2000 mg/l. A blood contamination of 0.1% (filled triangles) would raise the CSF concentrations to the following values, whereas the concentrations in blood would remain unchanged: albumin CSF 240 mg/l; IgG CSF 30 mg/l; IgA CSF 4 mg/l; IgM CSF 3 mg/l. A blood contamination of 1% (filled circles) would rise the CSF concentrations to the following values, whereas again the concentrations in blood would remain unchanged: albumin CSF 600 mg/l; IgG CSF 120 mg/l; IgA CSF 22 mg/l; IgM CSF 21 mg/l. Please note that blood contamination causes an increase in all quotients. The relative rise in the quotients increases with the size of the molecules studied. Even a low blood contamination can falsify an intrathecal synthesis of IgM. A blood contamination of 1% would seemingly lead to an intrathecal IgA and IgM synthesis. For these reasons, an intrathecal IgM synthesis in the presence of erythrocytes or hemoglobin in CSF must be interpreted with caution, and beyond a blood contamination 0.1% these nomograms should not be used

Many ventricular CSF specimens contain blood, either because of the underlying disease or as a consequence of the implantation of the ventricular catheter. When dealing with blood in CSF samples in several clinical settings, it must be taken into account that blood can heavily disturb CSF analysis of routine parameters. Blood in the CSF can originate (a) from an intracranial spontaneous or traumatic hemorrhage; (b) from surgery including ventriculostomy insertion, or a traumatic lumbar (or suboccipital) puncture. Blood in the CSF usually is detected by counting erythrocytes or by measuring free hemoglobin [74]. It can affect almost all CSF routine parameters, in particular the CSF leukocyte and differential leukocyte count, the CSF protein and albumin concentration, the CSF-to-serum albumin ratio, and the Reiber–Felgenhauer nomograms to detect intrathecal synthesis of IgG, IgA or IgM. In particular, the estimation of the intrathecal IgM synthesis is rapidly affected by blood contamination of the CSF (Fig. 3).

Whether CSF findings should be corrected for blood contamination and if so, by which formula, is a matter of debate. The easiest correction method is based on the assumption of a density of 5 × 10^6^/μl erythrocytes and 5 × 10^3^/μl leukocytes, i.e., a leukocyte/erythrocyte quotient of 1/1000 in blood: 5000 erythrocytes/μl CSF indicate a blood contamination of 0.1%. For the assessment of inflammation 1 leukocyte/μl is subtracted from the CSF white blood cell count for each 1000 erythrocytes/μl counted. The main advantages of this method of correction are: (1) It is easy to perform and (2) the laboratory analyzing the CSF does not need to know the true leukocyte and erythrocyte densities (which often are unavailable in specialized CSF laboratories) in the patient`s blood. A more sophisticated method using the variable leukocyte/erythrocyte quotient in blood is the cell index proposed by B. Pfausler and colleagues [69]. Here, the quotient of leukocytes to erythrocytes in CSF divided by the quotient of leukocytes to erythrocytes in peripheral blood is calculated. The cell index as an indicator for a CNS infection, however, does not account for the inflammatory stimulus caused by blood in the CSF space leading itself to the migration of leukocytes into the CSF [29]. Moreover, the cell index assumes that leukocytes and erythrocytes are homogeneously distributed in the CSF space. Since leukocytes and erythrocytes possess different densities, they separate during gravity sedimentation with leukocytes positioned on top of the erythrocytes [29, 75] (Fig. 2). Finally, granulocytes and monocytes can lyse or phagocytose erythrocytes [76], which also alters the ratio of leukocytes and erythrocytes, particularly when the entry of blood into the CSF occurred several days before the collection of the CSF specimen. For these reasons, a true estimate of blood contamination of CSF by counting erythrocyte density is likely not possible, and the cell index thus has only spurious accuracy. For the diagnosis of bacterial meningitis, correction for blood contamination by several methods did not improve the predicting algorithm. As there was no difference between AUC values for corrected and uncorrected leukocytes, adjusted blood counts in CSF appeared to have no advantage over uncorrected counts for predicting bacterial meningitis [77].

## Variation of CSF routine parameters

### Temporal variation

#### Development and resolution of inflammation

In animal experiments, it was found to take at least 12 h after the entry of bacteria for leukocytes to migrate into the CSF, and at least 15 h until bacterial meningitis fully developed [78]. For this reason, early in the course of bacterial meningitis, particularly in meningococcal meningitis (and pneumococcal meningitis in splenectomized and otherwise immunocompromised patients), the causative bacterium can be grown in the CSF, whereas the CSF leukocyte density and the CSF protein content are normal or slightly elevated (Table 1) [79]. Conversely, when the pathogen is eliminated, inflammation often resolves slowly [80, 81]. After successful treatment of bacterial meningitis, an increase in the inflammatory response in the CSF within the first 24 h is sometimes observed as a consequence of bacterial lysis [82, 83]. Thereafter, CSF leukocyte counts slowly decline, and an elevated CSF leukocyte density can be seen for up to several months after successful treatment of bacterial meningitis or another CNS infection [81, 82, 84]. Usually the decrease in CSF lactate is more rapid than the decrease in the CSF leukocyte or protein content [81, 84].

**Table 1:** Frequent pitfalls in the cerebrospinal fluid analysis in central nervous system infections

CSF parameter	Condition
CSF leukocyte count higher than expected	Blood contamination by underlying disease or traumatic puncture
CSF leukocyte count lower than expected	Leukocytopenia in the systemic circulation Early bacterial meningitis Rapid leukocyte death as a consequence of very high CSF concentration of bacteria Resolution of inflammation during adequate antibiotic therapy Sedimentation of leukocytes into bottom parts of the CSF space by gravity Analysis of ventricular instead of lumbar CSF
CSF erythrocyte count higher than expected	Previously unrecognized subarachnoid hemorrhage Prior surgery Traumatic puncture
CSF erythrocyte count lower than expected	Clotting of erythrocytes Sedimentation of erythrocytes into bottom parts of the CSF space by gravity Analysis of ventricular instead of lumbar CSF Phagocytosis of erythrocytes by invading macrophages and granulocytes
CSF lactate higher than expected	Meningeosis neoplastica Intracranial hemorrhage Other severe diseases inducing anaerobic glycolysis in the CNS
Unexpected increase of the CSF–serum albumin ratio	Old age Elevated body weight Genetic and environmental factors Height Female sex High ventricular volume High volume of the cerebral subarachnoid space Narrow spinal canal, spinal disc prolapse, Stenosis of the spinal canal Ventriculoperitoneal shunt Rapid correction of an intravascular volume deficit Severe blood loss with volume substitution by electrolyte solutions Severe albumin loss, e.g., after ascites puncture
Unexpected intrathecal synthesis of immunoglobulins in Reiber–Felgenhauer nomograms	CSF blood contamination (sensitivity: IgM > IgA > IgG) Removal of circulating immunoglobulins, e.g., by immune absorption Albumin intravenous infusion (temporary lowering of CSF-to-serum albumin ratio)
False-negative intrathecal synthesis of immunoglobulins in Reiber–Felgenhauer nomograms	High-dose intravenous infusion of immunoglobulins (temporary lowering of the CSF-to-serum immunoglobulin ratios)
Normal pathogen-specific antibody index (AI)	Does not rule out early infection, because in the first days no or very little pathogen-specific antibodies are produced
High pathogen-specific antibody index (AI)	A high pathogen-specific AI does not necessarily indicate acute infection, because after successful treatment or spontaneous recovery the decline of the pathogen-specific IgG or IgM concentrations in serum often is quicker than in CSF increasing the AI during reconvalescence

**Fig. 4:**
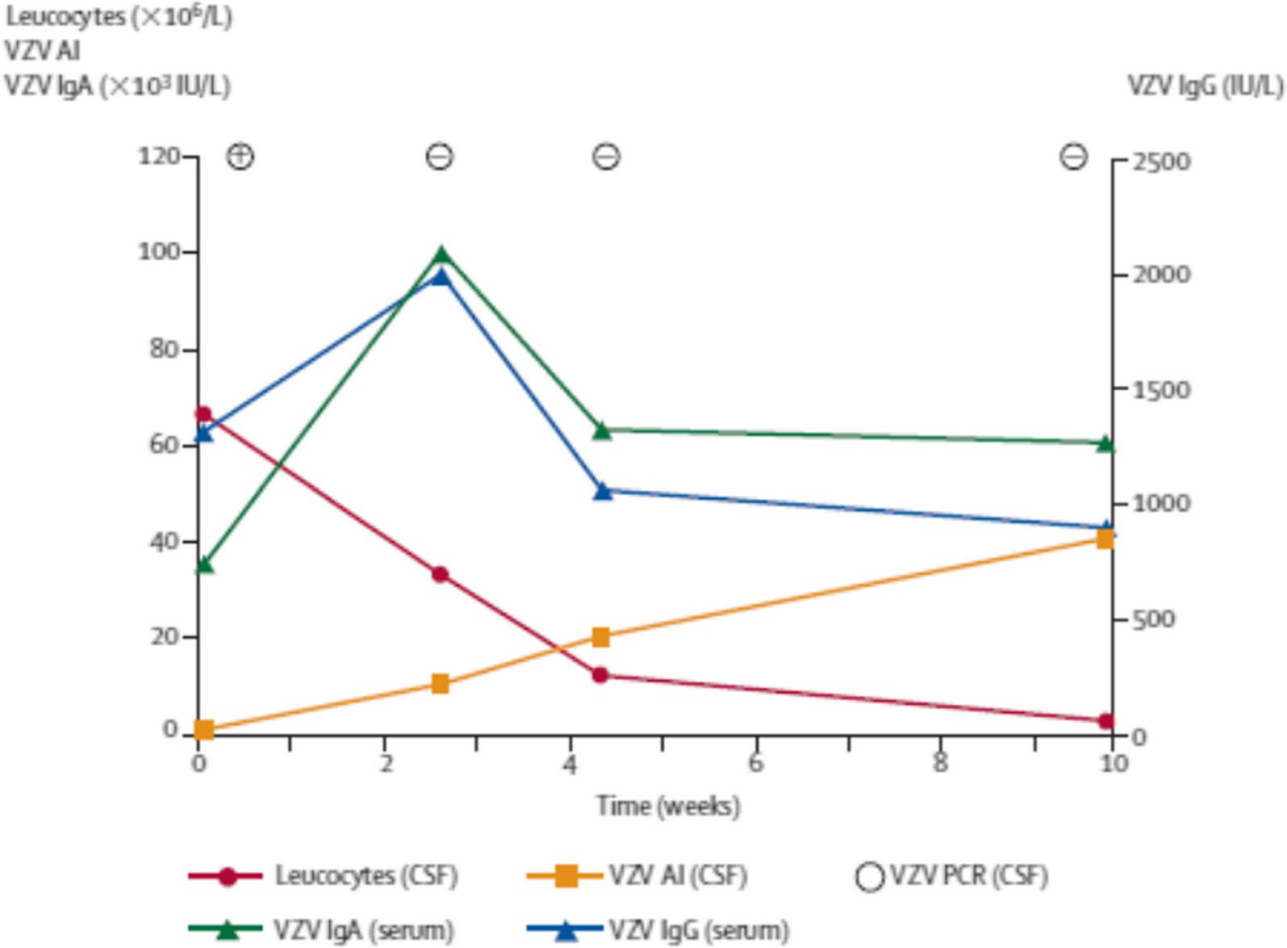
Increase in the pathogen-specific antibody index (AI) for IgG after successful treatment of CNS infections. In a 66-years old patient with Varicella zoster virus (VZV) cerebellitis at the first lumbar puncture, when VZV DNA was detected in CSF by nucleic acid amplification (PCR), the VZV-AI was not elevated. After successful treatment with aciclovir, the VZV-PCR became negative. The patient almost completely recovered, whereas the VZV-AI steadily rose [86]. Reproduced with kind permission of Elsevier HCM—Health Content Management

The pathogen-specific antibody index (AI), calculated for several classes of immunoglobulins (Ig, usually IgG or IgM) as:

$${\text{Antibody}}\;{\text{index}}\;{\text{(AI) }} = \frac{{\frac{{{\text{Pathogen-specific}}\;{\text{Ig}}\;{\text{in}}\;{\text{CSF}}\;{\text{(U}}/{\text{ml}})}}{{{\text{Pathogen-specific}}\;{\text{Ig}}\;{\text{in}}\;{\text{serum}}\;{\text{(U}}/{\text{ml)}}}}}}{{\frac{{{\text{Total}}\;{\text{Ig}}\;{\text{in}}\;{\text{CSF}}\;{\text{(mg}}/{\text{l}})}}{{{\text{Total}}\;{\text{Ig}}\;{\text{in}}\;{\text{serum}}\;{\text{(mg/l)}}}}}},$$

is a useful method to diagnose CNS infections and autoimmune diseases of the CNS [16, 85]. After successful treatment of a CNS infection, the concentrations of pathogen-specific antibodies often decrease faster in serum than in CSF. This leads to the paradoxical situation that the pathogen-specific AI increases, whereas both the serum and CSF concentrations of the pathogen-specific antibodies decrease [86] (Fig. 4) (Table 1). Attempts have been made to discriminate between an intrathecal immunoglobulin synthesis during CNS infections and autoimmune CNS diseases based on the absolute concentrations of pathogen-specific antibodies or the fraction of intrathecally produced pathogen-specific immunoglobulins of the total intrathecally produced immunoglobulins in CSF [16, 87]. The pathogen-specific AI can be elevated for years, even when all other signs of inflammation in the CSF have resolved [88, 89].

#### Short-term temporal variation: repeated CSF analyses from the same region

In a prospective study in patients with external ventriculostomy and with sterile CSF, sampling of ventricular CSF was followed by a 2nd sample 10 min later. During these 10 min either a standard clinical re-positioning was performed (the patient was positioned in a lateral side position for 2 h prior to sample 1, then the patient was re-positioned to the contralateral side prior to the collection of the 2nd sample; R-samples), or patients were not moved for 2 h before the 1st sampling and during the 10 min interval between 1st and 2nd sampling (control = C-samples). In 162 pairs of CSF samples from 51 patients, both re-sampling and re-positioning influenced CSF leukocyte and erythrocyte densities: the effect of re-positioning was stronger than the effect of re-sampling with a constant head position, the direction of change was random, variability increased with the leukocyte and erythrocyte count in sample 1, and leukocytes, granulocytes and erythrocytes showed greater variability than lactate or glucose [29]. When the Stockholm external ventricular drain related infection (EVDI) criteria for suspected EVDI were used, 14 2nd R-samples changed the EVDI diagnostic group, whereas no changes of the diagnostic group were observed with pairs of C-samples [29]. The authors concluded that the surveillance of CSF leukocytes and erythrocytes including methods of correction for blood contamination were unreliable for early detection of EVDIs.

#### Short-term variations of bioactive compounds

In a microminipig, after placement of a ventricular drain into the cisterna magna, melatonin in CSF showed a ≥ tenfold higher value at night than during the day, whereas no substantial diurnal variation in the CSF lactate dehydrogenase, creatine kinase, glucose, sodium, potassium, chloride, calcium, glutamine or alanine concentrations were noted. Peak CSF melatonin concentrations at night were 2–3 times higher than the respective blood concentrations during the day. CSF leukocytes, protein and lactate were not determined [90]. This study illustrates that the concentration of some hormones which use the CSF as communication channel can fluctuate rapidly. However, we are not aware of any related studies in humans.

### Spatial variation: ventricular versus cisternal versus lumbar CSF

#### Uninflamed meninges

Since it would be unethical withdraw CSF without a clear indication, we are unaware of comparisons of the composition of ventricular, cisternal and lumbar CSF in healthy volunteers after simultaneous ventricular, cisternal and lumbar puncture. In clinical routine CSF analysis, the normal values of the CSF-to-serum albumin ratio and of the total protein concentration in ventricular CSF are considered to be 2.5 times lower than the normal lumbar CSF values [18, 85]. In human ventricular CSF normal values are largely unknown. “Normal” values often are assumed in the ventricular CSF of patients requiring ventriculoperitoneal shunt surgery or an external ventricular drain for non-inflammatory hydrocephalus.

In an evaluation of routine data from a large neurochemical laboratory using mainly ventricular and lumbar CSF from different patients (i.e., not pairs of CSF samples from the same patient), the concentrations of proteins originating from neurons or glial cells (tau protein, neuron-specific enolase, S-100 protein) decreased from ventricular to lumbar CSF. In contrast, concentrations of blood-derived proteins and primarily leptomeningeal proteins (beta-trace protein and cystatin C) increased from ventricular to lumbar CSF [17].

Since cisternal CSF samples are very rare in clinical routine due to the risk of damaging the brain stem by suboccipital puncture, we attempted to collect samples representing an approximation to cisternal CSF in patients with clinical and radiological signs of normal pressure hydrocephalus by serial sampling of 40 ml in 5 ml fractions. Fraction 1 represented lumbar CSF, whereas fraction 8 was assumed to primarily contain CSF from regions close to the cisterna magna and cisterna pontis [91]. Normal CSF leukocyte counts and lack of intrathecal antibody synthesis in Reiber–Felgenhauer nomograms indicated an absence of inflammation. Median total CSF protein decreased from 433 mg/l (fraction 1) to 353 mg/l (fraction 8), and CSF albumin declined from 253 mg/l to 202 mg/l. Lumbar and “cisternal” CSF protein and albumin concentrations were strongly correlated (Spearman`s rank correlation coefficient *r*_S_ 0.95 and 0.91; *p* < 0.0001 each, *n* = 16 pairs of samples). As expected for brain-derived proteins, median tau, phosphorylated tau (ptau) and β-amyloid_1-42_ concentrations were slightly lower in lumbar than in “cisternal” CSF (*p* < 0.05 for ptau, *p* > 0.05 for tau), whereas this gradient was absent for β-amyloid_1-42_ [91]. A similar rostro-caudal decrease was described for α-synuclein and neuron-specific enolase in serial samples from 5 patients with normal pressure hydrocephalus. In these samples, the meningeal β-trace protein, as expected, showed a slight rostro-caudal increase [28]. As expected, the ventricular–lumbar concentration gradients in normal pressure hydrocephalus patients for tau and ptau were higher than the cisternal–lumbar gradients (ratios approximately five- and twofold) [92–94].

In a large study, analyzing uninflamed CSF from different patients (i.e., no pairs of CSF samples), the median ventricular CSF-to-serum glucose ratio was 0.70 (5th–95th percentile: 0.48–0.93), and the median lumbar ratio was 0.60 (5th–95th percentile: 0.41–0.81). There was thus a small ventriculo-lumbar decrease, which failed to reach statistical significance in a multivariate linear regression analysis [95].

**Fig. 5:**
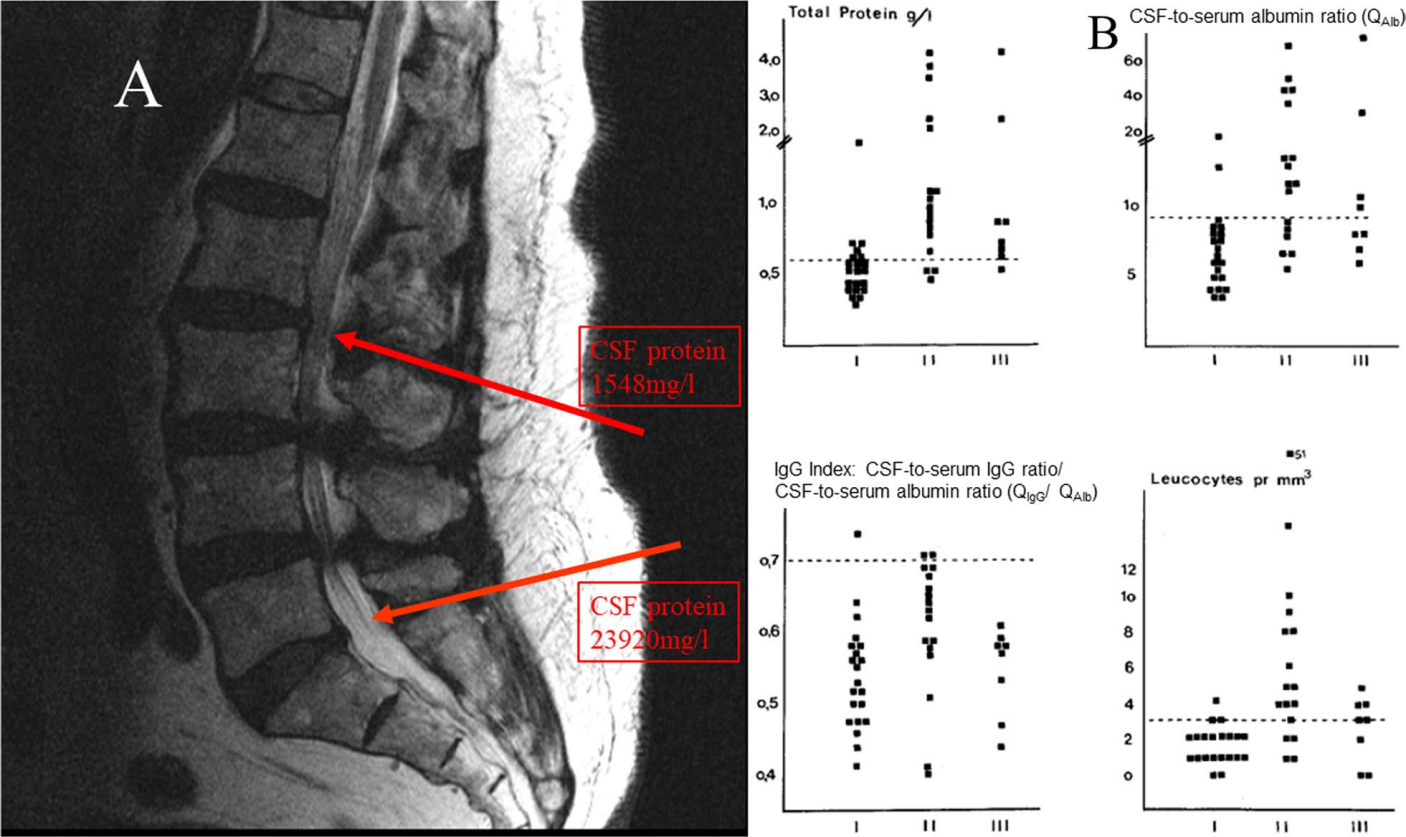
Increase in the total protein content and of the CSF-to-serum albumin concentration ratio (*Q*_Alb_) in lumbar CSF during disturbances of CSF circulation. **A** CSF findings cranial and caudal of two disc protrusions (T2-weighted magnetic resonance imaging, puncture sites highlighted by red arrows) (kindly provided by Dr. Hans-Heino Rustenbeck, Dept. of Neuroradiology, University Medicine Göttingen, Germany). First lumbar puncture between 5th lumbar vertebra and Os sacrum: total protein 23920 mg/l, *Q*_Alb_ 338 × 10^–3^, lactate 2.6 mmol/l; second lumbar puncture between 2^nd^ and 3rd lumbar vertebrae: total protein 1548 mg/l, *Q*_Alb_ 19.7 × 10^–3^, lactate 1.6 mmol/l. **B** Lumbar CSF findings in patients with normal pressure hydrocephalus (NPH) prior and after placement of a ventriculoperitoneal shunt (I—prior; II—3 months; III—year after implantation of a ventriculoperitoneal shunt [96]. After shunting, the lumbar CSF represents a backyard of CSF circulation. Reproduced with kind permission of Springer Nature

In normal pressure hydrocephalus, leukocyte count in lumbar CSF is normal (≤ 4/μl), and CSF-to-serum albumin ratio and CSF protein content are normal or slightly elevated. One year after placement of a ventriculoperitoneal shunt, in 7 of 8 patients leukocyte density in lumbar CSF was normal (≤ 4/μl), and in one patient it was 5/μl, whereas the lumbar CSF-to-serum albumin ratio and the lumbar CSF protein content had increased substantially [96] (Table 1) (Fig. 5). These observations suggest that in the absence of meningeal inflammation the cut-off of the CSF leukocyte count (4 μg/l) appears to remain valid even in the presence of a strongly elevated CSF protein content. Narrowing of the spinal canal also leads to an increase in the protein content in the CSF distal of the stenosis (Fig. 5). Since the exchange of CSF is strongly reduced caudal of the stenosis, the elevation of the CSF protein content or the CSF-to-serum albumin ratio depends on the narrowing of the spinal canal [97].

#### Meningeal inflammation

In an infant rhesus monkey model of *Haemophilus influenzae* meningitis after intranasal infection, simultaneously determined mean ventricular and cisternal *H. influenzae* concentrations were almost equal (1.1 × 10^8^ CFU/ml versus 1.3 × 10^8^ CFU/ml, *n* = 17). Of 8 simultaneous comparisons between ventricular and lumbar CSF, the difference in 5 pairs of samples was ≤ 1 log CFU/ml, and in 2 of the 3 discordant pairs of CSF samples, bacterial densities in the ventricular were greater than in the lumbar CSF [98]. In experimental *H. influenzae* meningitis, CSF leukocyte concentrations in ventricular CSF ranged from 1 to 3980/μl. Of 20 simultaneous comparisons between ventricular and cisternal CSF, leukocyte concentrations were about equal in 5, but were lower in the ventricular than in the cisternal CSF in 15 pairs of samples (mean ventricular/cisternal ratio 0.08, range 0.0–0.20). CSF protein, glucose and lactate and CSF-to-serum albumin ratio were not determined [98].

In humans, differences in the composition between ventricular and lumbar CSF in CNS infections were first noted by Merritt and Freemont-Smith in 1937 [27]. In 3 patients with hydrocephalus and basal meningitis (2 cryptococcal meningitis, 1 tuberculous meningitis), leukocytes in ventricular CSF were normal (≤ 4/μl), and CSF protein ranged from 80–270 mg/l, whereas in lumbar CSF obtained 4–8 days later, CSF leukocytes ranged from 86 to 540/μl and CSF protein from 1010 to 11,780 mg/l. The authors concluded that a normal ventricular CSF does not rule out a bacterial or fungal CNS infection, particularly in patients presenting with hydrocephalus and basal meningitis [99]. Microbiological testing can diverge between ventricular and lumbar CSF: in one case, cryptococcal antigen was negative in ventricular and positive in lumbar CSF, and in another case, TBC PCR was positive in the ventricular CSF despite normal protein and leukocyte count, whereas in the lumbar CSF TBC PCR and culture were negative despite a strong elevation of CSF protein and leukocyte density [99]. The main limitation of this study, however, was the relatively long interval between analysis of ventricular and lumbar CSF.

In 41 pairs of samples from 26 patients with a suspected bacterial CNS infection (nosocomial or community-acquired), who received a lumbar and ventricular CSF analysis within 24 h, the correlation between ventricular and lumbar leukocyte count was lowest (Spearman`s rank correlation coefficient *r*_S_ = 0.37), between ventricular and lumbar total protein concentration intermediate (*r*_S_ = 0.42), and highest between ventricular and lumbar lactate concentrations (*r*_S_ = 0.79) [11]. On average the lumbar concentrations were higher. However, in 7 pairs the ventricular was higher than the lumbar leukocyte density, in 5 pairs the ventricular was higher than the lumbar protein concentration, and in 8 pairs the ventricular was higher than the lumbar lactate concentration [11]. It was concluded that “findings typical for bacterial meningitis in one part of the CSF compartment may be absent in another” [11].

**Fig. 6:**
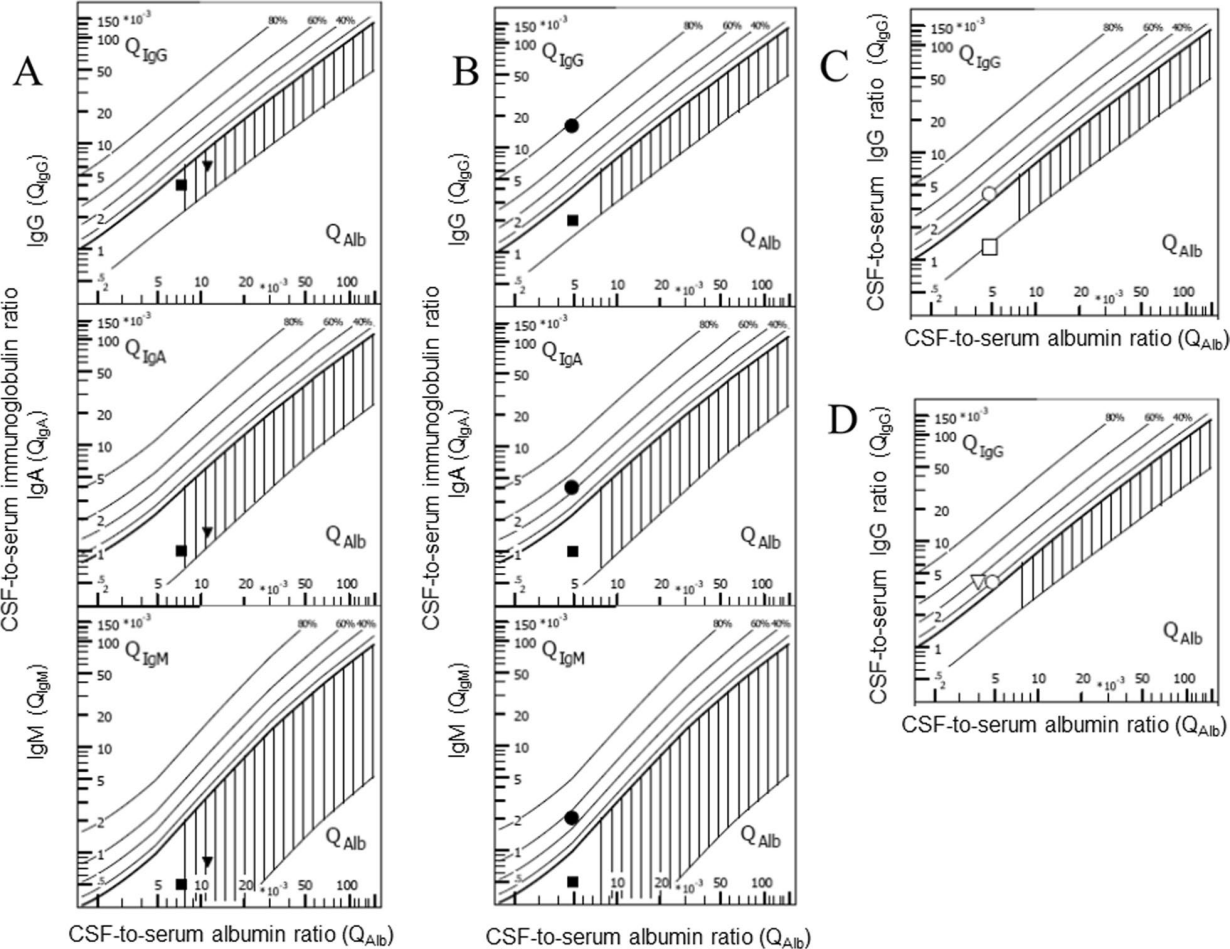
Limitations of the Reiber–Felgenhauer nomograms in the absence of steady state—hypothetical cases. In each case the values used for the calculation of the quotients are given. **A** Seeming impairment of the blood–CSF barrier in a patient after rapid correction of an intravascular volume deficit of 33% (filled squares: albumin CSF 300 mg/l, serum 40,000 mg/l, IgG CSF 40 mg/l, serum 10,000 mg/l, IgA CSF 2 mg/l, serum 2000 mg/l, IgM CSF 1 mg/l, serum 2000 mg/l; filled reverse triangles: albumin CSF 300 mg/l, serum 26,700 mg/l, IgG CSF 40 mg/l, serum 6660 mg/l, IgA CSF 2 mg/l, serum 1330 mg/l, IgM CSF 1 mg/l, serum 1330 mg/l). **B** Seeming intrathecal antibody synthesis after removal of 75% of the intravenous immunoglobulins by immune absorption (before immunoadsorption—filled squares: albumin CSF 200 mg/l, serum 40,000 mg/l, IgG CSF 20 mg/l, serum 10,000 mg/l, IgA CSF 2 mg/l, serum 2000 mg/l, IgM CSF 1 mg/l, serum 2000 mg/l; after immunoadsorption—filled circles: albumin CSF 200 mg/l, serum 40,000 mg/l, IgG CSF 40 mg/l, serum 2500 mg/l, IgA CSF 2 mg/l, serum 500 mg/l, IgM CSF 1 mg/l, serum 500 mg/l). **C** Apparent disappearance of an intrathecal IgG synthesis after infusion of 120 g IgG (before IgG infusion—open circles: albumin CSF 200 mg/l, serum 40,000 mg/l, IgG CSF 40 mg/l, serum 10,000 mg/l, IgA CSF 2 mg/l, serum 2000 mg/l, IgM CSF 1 mg/l, serum 2000 mg/l; after IgG infusion—open squares: albumin CSF 200 mg/l, serum 40,000 mg/l, IgG CSF 40 mg/l, serum 30,000 mg/l, IgA CSF 2 mg/l, serum 2000 mg/l, IgM CSF 1 mg/l, serum 2000 mg/l). **D** Seeming increase in intrathecal IgG synthesis after infusion of 60 g albumin (before albumin infusion—open circles: albumin CSF 200 mg/l, serum 40,000 mg/l, IgG CSF 40 mg/l, serum 10,000 mg/l, IgA CSF 2 mg/l, serum 2000 mg/l, IgM CSF 1 mg/l, serum 2000 mg/l; after albumin infusion—open reverse triangles: albumin CSF 200 mg/l, serum 50,000 mg/l, IgG CSF 40 mg/l, serum 10,000 mg/l, IgA CSF 2 mg/l, serum 2000 mg/l, IgM CSF 1 mg/l, serum 2000 mg/l)

**Fig. 7:**
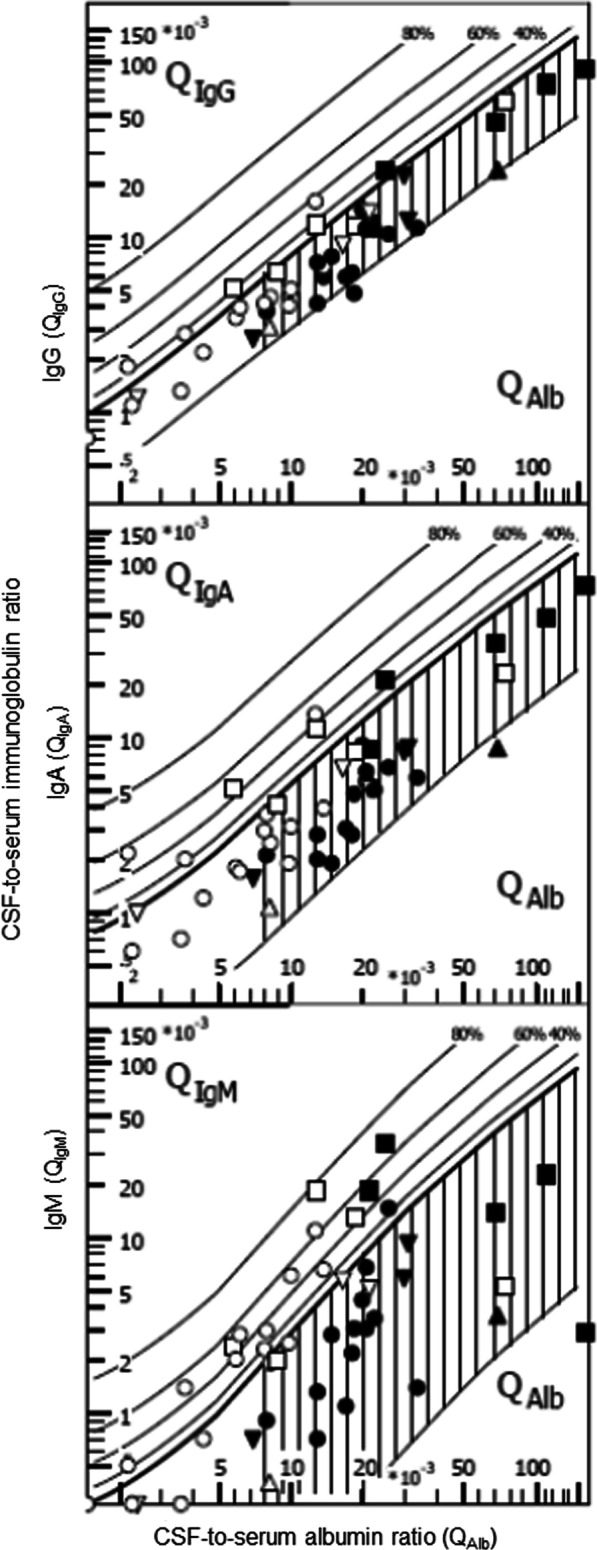
Re-analysis of findings from patients receiving a lumbar and ventricular CSF puncture within 30 min [100]. Filled symbols represent lumbar, open symbols ventricular CSF parameters. Different diseases are indicated by different symbols—squares: bacterial meningitis; circles: cerebral/ventricular hemorrhage; triangles reverse: cerebral infarctions; triangles: meningeosis carcinomatosa. Intrathecal immune globulin synthesis was most often detected in the case of IgM, and was more frequent in ventricular than in lumbar CSF. One probable reason for these findings is the lower CSF-to-serum albumin ratio (*Q*_Alb_) in ventricular CSF, which increases the susceptibility of this analytical procedure for blood contamination (for details see text)

Because the interval of ≤ 24 h between the analysis of lumbar and ventricular CSF is rather long in view of the rapid temporal changes occurring in CNS infections, a prospective study was performed with an interval between lumbar and ventricular sampling < 30 min in 25 patients (15 intracranial hemorrhage, 6 bacterial CNS infections, 3 cerebral infarctions, 1 meningeosis carcinomatosa) [100]. Again, the correlation between lumbar and ventricular concentrations was lowest for CSF leukocyte density (*r*_S_ = 0.455), intermediate for CSF total protein and albumin (*r*_S_ = 0.624 and 0.525), and highest for the CSF lactate concentration (*r*_S_ = 0.767). The correlation of the lumbar versus ventricular erythrocyte density (*r*_S_ = 0.224) was even lower than the correlation between lumbar and ventricular total leukocyte density and the percentages of the differential white blood cell counts (*r*_S_ 0.271–0.644). On average, glucose concentrations were slightly higher in the ventricular than in the lumbar CSF, and lactate and erythrocyte density were approximately equal. The lumbar concentrations of albumin, total protein, IgG, IgA, IgM, and lumbar leukocyte densities were higher than the ventricular concentrations [100]. These differences remained similar, irrespective of whether hemorrhage or infection was studied. With all parameters studied, the ventricular was higher than the lumbar concentration in at least one pair of samples. The re-analysis of CSF-to-serum ratios of albumin, IgG, IgA and IgM in Reiber–Felgenhauer nomograms, suggested in some cases an intrathecal antibody synthesis. This probably was a consequence of the strong blood contamination in most samples (Figs. 6, 7).

A recent study on 12 patients with an external ventriculostomy and an external lumbar drain (7 intracranial hemorrhages, 3 infections, 1 tumor, 1 vascular malformation) analyzing pairs of samples obtained at an interval < 24 h found a high correlation between cranial and spinal CSF leukocyte density (*r* = 0.944), protein (*r* = 0.679) and glucose (*r* = 0.805), whereas no statistically significant correlation of the ventricular and lumbar erythrocyte density was found. Again, on an average, the ventricular was higher than the lumbar glucose concentration, and the lumbar protein and leukocyte concentrations were higher than the respective ventricular concentrations. A reversal of these gradients, however, was present in at least one pair of samples [101]. In 3 of 4 patients with positive CSF cultures, bacteria grew only in either the ventricular or the lumbar CSF [101]).

In 16 patients with hydrocephalus as a consequence of neurocysticercosis, who received a ventriculoperitoneal shunt and a lumbar puncture at an interval of approximately 30 min, mean white blood cell density was 14 ± 5/μl in the ventricular and 72 ± 28/μl in the lumbar fluid. The protein content in the ventricular CSF was 280 ± 50 mg/l (mean ± SD) compared to 780 ± 120 mg/l in the lumbar CSF. Immunodiagnostic tests for cysticerci antigens (enzyme immunoassay and complement fixation) were positive in 12 of 16 ventricular and in all 16 lumbar samples. Again, glucose was higher in the ventricular than in the lumbar CSF [102].

### Pitfalls in the interpretation of Reiber–Felgenhauer nomograms

#### Influence of a disequilibrium between blood and CSF

In contrast to albumin, which is synthesized by the liver, immunoglobulins can be synthesized by B-lymphocytes in the systemic circulation or in the CNS. For the detection of an inflammatory process involving immunoglobulin synthesis in the CNS it is insufficient to merely measure CSF immunoglobulin concentrations. For this reason, nomograms using CSF/plasma ratios for albumin and immunoglobulins in persons without diseases of the nervous system were established as references to discriminate between local immunoglobulin synthesis in the CNS and an increase in CSF protein concentration for other reasons [103]. In contrast to earlier attempts, the nomograms developed by Reiber & Felgenhauer relying on the CSF-to-serum quotients of albumin (to quantify the state of the blood–CSF barrier) and IgG, IgA and IgM possess a high specificity for detecting and quantifying the synthesis of IgG, IgA or IgM in the CNS [85, 104]. An important prerequisite for the use of these nomograms, however, is an approximate steady state (as a consequence of the high intra-individual variation of various physiological parameters, including the CSF production rate, an exact steady state can be never reached). Applying these nomograms soon after a severe disturbance of the steady state may lead to wrong conclusions (Fig. 6).

#### Influence of artificial or true blood admixture in CSF on Reiber–Felgenhauer nomograms

The Reiber–Felgenhauer nomograms are susceptible to errors, when the CSF is contaminated by blood (Table 1). As shown in Fig. 3, the nomogram of IgM is most susceptible to blood contamination, followed by the nomograms of IgA and IgG. As already mentioned, an exact estimation of blood contamination is not possible, and thus the clinician interpreting the results after a lumbar or ventricular puncture must be aware that even a mild blood contamination of the CSF can simulate an intrathecal IgM synthesis [85]. In pairs of lumbar and ventricular CSF samples drawn at an interval < 30 min (*n* = 25) [100], the erythrocyte concentrations in lumbar CSF ranged from 0 to 180,000/μl and in ventricular CSF from 5 to 80,000/μl. These data were re-analyzed by the program “Protein Statistics in CSF Analysis with Reibergrams” (by Werner Albaum with Prof. Hansotto Reiber, Albaum IT-Solutions, Göttingen, Germany, Version 4.17, 2012–2013). The Reiber–Felgenhauer nomograms of lumbar CSF suggested intrathecal IgM synthesis in 3 cases, IgA synthesis in 1 case, and IgG synthesis in 1 case (Fig. 7). The Reiber–Felgenhauer nomograms of ventricular CSF indicated intrathecal IgM synthesis in 11 cases, IgA synthesis in 5 cases, and IgG synthesis in 5 cases (Fig. 7). In 3 cases, intrathecal synthesis was comparable in lumbar and ventricular CSF. One of these was a patient with tuberculous meningitis who displayed intrathecal synthesis of IgG, IgA and IgM both in lumbar and ventricular CSF. In 8 cases, intrathecal synthesis was only detected in ventricular CSF (IgG, IgA and IgM: 4 cases; IgM only: 4 cases). Intrathecal immunoglobulin synthesis in lumbar CSF only was not observed in any patient. A chronic inflammatory process (tuberculous meningitis) was present in only 2 of the 25 cases (one with IgG, IgA and IgM synthesis in lumbar and ventricular CSF, one without intrathecal immunoglobulin synthesis). Acute meningitis or meningoencephalitis was diagnosed in 4 cases (one with intrathecal synthesis of IgG, IgA and IgM detected in ventricular CSF only, one with an intrathecal IgM synthesis both in lumbar and ventricular CSF, 2 without detectable intrathecal immunoglobulin synthesis). Meningeosis carcinomatosa was diagnosed in 1 case without detectable intrathecal immunoglobulin synthesis. In all but one patient (IgM, bacterial meningitis), the percentage of intrathecal synthesis of all immunoglobulins estimated by Reiber–Felgenhauer nomograms was greater in ventricular than in lumbar CSF (Fig. 7). In 9 of 18 patients diagnosed with cerebrovascular diseases, intrathecal immunoglobulin synthesis was detected (in 6 IgM only). In none of these patients was the CSF without erythrocytes, and 5 had erythrocyte counts ≥ 5000/μl in lumbar or ventricular CSF. This suggests that in a high proportion of the patients with cerebrovascular diseases, what seemed to be intrathecal immunoglobulin synthesis was rather a consequence of blood contamination. Because of the problems discussed above, in our lab we do not attempt to correct the Reiber–Felgenhauer nomograms for possible blood contamination of CSF. At erythrocyte densities > 5000/μl, i.e., at approximate blood contaminations > 0.1%, the nomograms are not used [85]. The problems of estimation of the true blood contamination by counting the erythrocytes in CSF are discussed above.

## The special case of lactate as a sensitive indicator of bacterial or fungal CNS infection

Lactate is a small hydrophilic molecule (molecular mass 89 g/mol). As outlined, it distributes more evenly in the CSF than larger molecules in health and disease [11]. Its concentration rapidly changes during the onset and the resolution of a bacterial or fungal infection of the CNS [84]. CSF lactate concentrations ≥ 3.5 mmol/l are considered typical for bacterial or fungal meningitis or meningoencephalitis [85]. Although many bacteria are able to produce lactate, over 95% of the CSF lactate concentration in bacterial meningitis originates from the host cells [105]. Host cells exclusively produce l-lactate, whereas some bacterial strains causing meningitis can produce d-lactate. Since even in experimental meningitis caused by d-lactate-producing bacteria the d-lactate fraction in CSF was below 5% [105], l-lactate is usually measured in the clinical routine. As a consequence of anaerobic energy production in the CNS, under normal conditions the CSF levels of lactate are higher than the respective serum concentrations [85, 105, 106]. Since the permeability of the blood–CSF and blood–brain barrier to anionic compounds such as lactate is low and apparently no active transport system for lactate exists at the blood–brain and blood–CSF barrier, after an intravenous infusion of lactate in non-human primates no increases in CSF lactate concentrations were observed [107]. In experimental meningitis, CSF lactate did not depend on the respective serum concentrations [106]. In a recent prospective observational cohort study aiming at the discrimination of community-acquired bacterial meningitis and aseptic meningitis/encephalitis in adults, of the CSF parameters lactate, leukocytes, fraction of neutrophils, protein and CSF-to-serum glucose ratio, CSF lactate had the best discriminatory value: its area under the receiver operating characteristic curve (AUROC) was 0.976 (95%CI 0.966–0.997). Using a cut-off of 3.5 mmol/L, its sensitivity was 96% and its specificity 85%. Antibiotic treatment before lumbar puncture had no significant effect on the AUROC of CSF lactate [108]. Lactate was also useful to distinguish between post-neurosurgical bacterial meningitis and aseptic meningitis: here, a lactate cut-off of 3.45 mmol/l yielded a sensitivity of 90% and a specificity of 85% [109]. In a meta-analysis on 33 studies comprising 1885 patients, a CSF lactate of 35 mg/dl (= 3.93 mmol/l) was proposed as optimum cut-off value in order to distinguish between bacterial and aseptic meningitis [110].

Since under anaerobic conditions the brain and CSF leukocytes produce lactate, lactate is not a specific marker of bacterial or fungal infection, but may be elevated in various conditions with elevated ICP or other causes of reduced cerebral perfusion. In 51 patients with subarachnoid hemorrhage and an external ventriculostomy, CSF lactate ranged from 1.9 to 6.2 mmol/L (median 3.2 mmol/l) [111]. The lactate concentration of ventricular and cisternal CSF in subarachnoid hemorrhage was approximately equal and depended on the severity of the hemorrhage [ventricular CSF, grade 1–2 of the World Federation of Neurosurgical Societies: 21.8 ± 7.0 mg/dl (2.4 ± 0.8 mmol/l), grade 3–5: 35.8 ± 18.8 mg/dl (4.0 ± 2.1 mmol/l); cisternal CSF, grade 1–2: 25.1 ± 5.5 mg/dl (2.8 ± 0.6 mmol/l), grade 3–5: 32.6 ± 7.4 mg/dl (3.7 ± 0.8 mmol/l)]. A high CSF lactate and a lactate increase were correlated with a poor outcome [112]. In 158 children with a CSF lactate concentration ≥ 2 mmol/l without CNS infections, the following diseases were noted: respiratory chain disorders, cerebral ischemia, malignant diseases and seizures [113].

During the course of bacterial meningitis, the most important CSF parameter predicting therapeutic efficacy or failure are the CSF-to-serum glucose ratio and CSF lactate concentrations [84]. The CSF white blood cell count and differential counts are much weaker predictors of therapeutic efficacy, since CSF pleocytosis may persist for weeks in spite of effective antibiotic therapy [84].

Compared to CSF lactate, CSF glucose possesses disadvantages: (1) The CSF lactate concentration is less dependent on the respective serum concentration than the CSF glucose concentration. Therefore, often the CSF-to-serum glucose concentration ratio is used instead of the CSF glucose level. (2) Serum glucose may rapidly change dependent on carbohydrate intake and insulin therapy. This probably impairs the stability of the CSF-to-serum glucose concentration ratio. (3) Unlike lactate, CSF glucose (and the CSF-to-serum glucose concentration ratio) possess a small, but not negligible ventricular–lumbar gradient.

## Conclusions

Temporal and spatial variations of CSF parameters and an impaired immune response can complicate the etiological diagnosis of CNS infections based on the CSF routine parameters leukocyte count, differential white blood cell count, protein, lactate, glucose and the CSF-to-serum rations of albumin, immunoglobulins and glucose. Patterns typical of certain infections may provide diagnostic hints, but are neither sensitive nor specific enough for effective clinical decision-makings. In clinical routine, the parameter which most readily equilibrates among the different parts of the CSF space, and has a relatively high sensitivity and specificity in the discrimination of bacterial/fungal versus viral meningitis and meningoencephalitis, is CSF lactate at a cut-off of 3.5(− 4) mmol/l. In severe non-infectious CNS diseases including intracranial hemorrhage, however, CSF lactate as a non-specific parameter of a suffering brain can also be elevated ≥ 3.5 mmol/l.

Thus, establishing a definite diagnosis of a CNS infection by clinical chemical or cytological routine parameters is still not possible at present. Diagnosis of infections relies on the identification of the causative pathogen by culture or molecular methods (e.g., 16 s rRNA amplification [114]; multiplex polymerase chain reaction [115]; metagenomic next-generation sequencing: [116]). Recent advances in molecular methods will open new avenues for the definite diagnosis of a variety of CNS infections, particularly in the presence of atypical or mild alterations of the composition of the CSF.

## Data Availability

Data sharing is not applicable to this article as no datasets were generated or analyzed during the current study.

## Abbreviations

AI: Antibody index
CSF: Cerebrospinal fluid
CCT: Cranial computer tomography
CNS: Central nervous system
IgA: Immunoglobulin A
IgG: Immunoglobulin G
IgM: Immunoglobulin M
MRI: Magnetic resonance imaging
Q_Alb_: CSF-to-serum albumin ratio
Q_IgG_: CSF-to-serum IgG ratio
Q_IgA_: CSF-to-serum IgA ratio
Q_IgM_: CSF-to-serum IgM ratio
PCR: Polymerase chain reaction
rRNA: Ribosomal ribonucleic acid
Tau: Tau protein
phospho-Tau: Phosphorylated Tau protein
VZV: Varicella zoster virus
